# Physiologically relevant media are associated with overlapping metabolic responses in primary human hepatocytes and Huh7 cells

**DOI:** 10.14814/phy2.70989

**Published:** 2026-07-03

**Authors:** Eloise Cross, Felix Westcott, Kieran Smith, Shilpa R. Nagarajan, Fabio Sanna, Kaitlyn M. J. H. Dennis, Leanne Hodson

**Affiliations:** ^1^ Radcliffe Department of Medicine, Oxford Centre for Diabetes, Endocrinology and Metabolism Churchill Hospital, University of Oxford Oxford UK; ^2^ National Institute for Health Research Oxford Biomedical Research Centre Oxford University Hospital Trusts Oxford UK

**Keywords:** in vitro, liver, metabolism, primary human hepatocytes

## Abstract

The initiation and progression of metabolic dysfunction‐associated steatotic liver disease (MASLD) is challenging to study in vivo in humans, and robust in vitro high‐fidelity disease models are limited. Although primary human hepatocytes (PHH) are often considered to be the gold standard, immortalized hepatic cell lines are often utilized because of their scalability and experimental tractability. Therefore, the aim of this study was to compare the metabolic responses of PHHs with our characterized Huh7‐based model when exposed to the physiologically relevant fatty acid (FA) mixtures. PHH and Huh7 cells were treated with 2% human serum and a combination of sugar and FAs enriched in either unsaturated (OPLA) or saturated (POLA) FAs for 4 or 7 days, respectively. Stable isotope tracers were utilized to investigate basal changes in hepatocyte metabolism in response to different treatment regimes. Changes in cell viability, media biochemistry, intracellular metabolism, lipid droplet morphology, and gene expression were quantified. Huh7 cells had greater viability than PHH, while NEFA uptake and triglyceride secretion were similar. Both OPLA and POLA increased the proportion of large lipid droplets in Huh7 cells, whereas only OPLA produced comparable effects in PHH. Despite higher baseline TG in PHH, both models showed similar lipid composition, de novo lipogenic responses, and glycogen levels. Compared to Huh7 cells, PHH exhibited higher media 3‐hydroxybutyrate, lower media lactate, reduced glucose uptake, and donor‐dependent transcriptomic variability. Our data demonstrate that Huh7 cells are metabolically adaptable and, when cultured in physiologically relevant media, metabolic readouts are more similar to those observed in PHH cells, thus making Huh7 a potentially useful workhorse model to investigate relevant pathways that may underpin the development of MASLD. Progress in MASLD research is constrained by limited access to human liver tissue and the scarcity of robust in vitro models. Although PHHs are considered the gold standard, they can be limited by poor viability and donor‐to‐donor variability. Huh7 cells are metabolically flexible and can recapitulate key metabolic responses of PHH when exposed to physiologically‐relevant chronic culture conditions, making them an alternative to PHH that is a scalable and reproducible model for investigating intrahepatic triglyceride (IHCTG) accumulation and MASLD progression.

## Graphical abstract

Overview of metabolic similarities and differences between Huh7 and primary human hepatocyte cells cultured in physiologically‐relevant media.

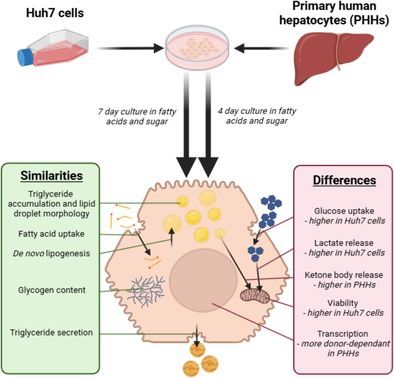

## 1 INTRODUCTION

Metabolic dysfunction‐associated steatotic liver disease (MASLD), which encompasses a spectrum of disease states from simple steatosis to steatohepatitis (Rinella et al., 2023), is initiated with the pathological accumulation of intrahepatocellular triglyceride (IHCTG) (Di Mauro et al., 2021). MASLD is estimated to affect approximately 30% of the global adult population (Younossi et al., 2023) and is an independent risk factor for cardiovascular disease (Jamialahmadi et al., 2023). Although it has been suggested that IHCTG accumulation starts to occur when there is an imbalance in hepatocyte fatty acid (FA) uptake, synthesis (de novo lipogenesis (DNL)) and disposal (i.e. as TG in VLDL or utilized in mitochondrial oxidation) (Babin & Gibbons, 2009; Diraison & Beylot, 1998; Hodson & Frayn, 2011; Sidossis et al., 1998), studying these processes in vivo in humans is challenging as high‐fidelity disease models are sparse.

The development and progression of MASLD in humans has been investigated through the use of liver biopsies, which offer valuable histological insights, but are invasive, can be difficult to obtain, limited to a single time point, and do not capture the temporal dynamics or spatial heterogeneity of disease progression. Although preclinical rodent models have been utilized to ascertain various aspects of the MASLD pathophysiology (Lau et al., 2017; Li et al., 2012), findings from Ding and colleagues (Ding et al., 2023) highlighted key divergences in the hepatic handling of FAs between humans and rodents, which may limit the translation of findings (Green et al., 2015). A variety of in vitro human cellular models have been utilized to gain insight into the mechanisms underpinning MASLD initiation and progression in vivo in humans; however, the usefulness of these models is questionable as their ability to recapitulate human disease is often limited (Green et al., 2015).

Primary human hepatocytes (PHH) and immortalized hepatic cell lines have been used as in vitro models of disease. PHH are often considered to be the gold standard (Green et al., 2015; Kwon et al., 2024; Younossi et al., 2023) as observations in PHH cells have not often been recapitulated in immortalized hepatocellular carcinoma cell lines, such as Huh7 and HepG2 (Huggett et al., 2022). However, PHH also come with challenges such as high costs, limited culturing capability, potential for low cell viability, and significant variation between donor phenotype and genotype (Godoy et al., 2013), making them somewhat burdensome for regular use when undertaking new methodologies and techniques or larger scale mechanistic experiments. The divergence in observations between PHH and cell lines may be due to non‐physiological media compositions and culturing processes, with in vitro cell line studies typically utilizing acute exposures (6–48 h) to supraphysiological concentrations of glucose, a single FA and/or other substrate treatments (Green et al., 2015). We (Gunn et al., 2017) and others (Pramfalk, Larsson, et al., 2016; Steenbergen et al., 2013, 2018) have previously reported that culturing human cell lines with human serum (HS), rather than foetal bovine serum (FBS), can alter the phenotype of cell lines to reflect improved functionality of IHCTG accumulation. Moreover, by exposing cell lines chronically (7 days of culturing) to more physiologically relevant substrate concentrations of FAs, sugars, and HS, our model displayed metabolic characteristics of early stage IHCTG accumulation (Gunn et al., 2020; Nagarajan et al., 2022). It remains unclear whether PHHs recapitulate these early‐stage characteristics when cultured in similar media conditions. Therefore, the aim of this study was to compare the metabolic responses of PHHs with our previously characterized Huh7‐based model (Nagarajan et al., 2022) when exposed to the physiologically relevant FA mixtures, OPLA (predominantly unsaturated) and POLA (predominantly saturated), as a model of early IHCTG accumulation. Given the differing viability and culture requirements of PHH and Huh7 cells, we compared each model under its optimal chronic culture conditions, rather than attempting perfectly matched exposure duration or basal media composition.

## 2 METHODS

### 2.1 Cell culture

Huh7 and PHH cells were used. Male Huh7 cells were kindly provided by Dr. Camilla Pramfalk (Karolinska Institute) and were cultured as previously described (Gunn et al., 2020; Nagarajan et al., 2022). PHH cells were obtained from Thermofisher (ThermoFisher, HMCPSQ (HU8339‐A, HU2016, HU8356, HU8354‐A, HU8148)) originating from both male and female donors (see Table S1 for donor characteristics). Frozen PHH cells were thawed in a 37°C water bath rapidly. 500 μL of warm plating media (500 mL Williams E Medium (ThermoFisher, A1217601)) supplemented with Primary Hepatocyte Thawing and Plating Supplements (ThermoFisher, CM3000) which contained FBS, dexamethasone, and a cocktail of penicillin–streptomycin, insulin, GlutaMAX™ and HEPES solution, was then added to the cryovial. Cells were then suspended and transferred to a falcon tube containing 4.5 mL of warm plating media. Cell number and viability were then quantified using Trypan blue stain (ThermoFisher, 15250061) and a Cellometer Auto T4 Bright Field Cell Counter. Cells were then diluted with plating media and seeded into plates which had been pre‐coated with collagen type I (Rat tail, ThermoFisher, A1048301) at a density of 35,000 cells/well for 96‐well plates, 150,000 cells/well for 24‐well plates or 50,000 cells/well for 12 mm diameter glass cover slips for microscopy experiments. Both Huh7 and PHH cells were maintained at 37°C in 5% CO_2_.

### 2.2 Experimental treatments

Huh7 cells were seeded into relevant plates and cultured in experimental media comprised of 2% HS, 5.5 mM glucose and 800 μM of 4 FAs (oleic acid (O, Sigma, O7501), palmitic acid (P, Sigma, P9767), linoleic acid (L, Sigma, L8134)) and alpha‐linoleic acid (A, Cambridge Biosciences, CAY21910) in one of two physiological ratios: OPLA (45:30:24:1) or POLA (44:45:10:1), or a no fat control as previously described (Nagarajan et al., 2022). Huh7 cells were cultured in experimental media for 7 days with media changes every other day as previously described (Nagarajan et al., 2022).

Following seeding, PHHs were cultured in plating media overnight then cultured in experimental media which consisted of Williams E Medium (ThermoFisher, A1217601) and Primary Hepatocyte Maintenance Supplements (ThermoFisher, CM4000) containing dexamethasone and a cocktail of penicillin–streptomycin, insulin, transferrin, selenium complex, BSA, GlutaMAX™, and HEPES solution, supplemented with 2% HS. PHH cells were cultured in 800 μM OPLA and POLA or no fat control experimental media for 4 days with media changes every other day.

### 2.3 Biochemical analysis

Media glucose, lactate, TG, apolipoprotein B (apoB0) and non‐esterified FA (NEFA) concentrations were measured on a AU480 chemistry analyzer (Beckman Coulter) and normalized to protein concentration as described (Gunn et al., 2017). Media ATP (Promega, G9681), acetate (Abcam, ab204719) and intracellular glycogen (Abcam BioVision, ab65620) were measured on the FLUOstar Omega (BMG, Labtech) according to the manufacturer's instructions.

### 2.4 Cell viability

We assessed cell viability in two ways. Prior to seeding in experimental media, Trypan Blue, which stains cells with an intact plasma membrane, was added to Huh7 and PHH cells. Cellular ATP concentrations were determined using the CellTiter‐Glo Luminescent Cell Viability Assay (Promega, G9681) according to manufacturer's instructions. Luminescence was read on FLUOstar Omega (BMG, Labtech). For this, we also assessed the effect of individual FAs and FA mixtures (i.e., OPLA and POLA) or a no‐fat control, on cell viability by culturing Huh7 and PHH cells in media supplemented with 400 μM oleate or 400 μM palmitate as either a single FA or as part of a FA mixture (800 μM OPLA or 800 μM POLA) with the media changed daily for 4 days. The amount of 400 μM of palmitic acid as a single FA was based on previous work (Boonkaew et al., 2025; Buratta et al., 2021; Seo et al., 2016) and allowed cell viability to be compared as our POLA FA mix contained the equivalent of 400 μM palmitic acid.

### 2.5 Lipid extraction and gas chromatography–mass spectrometry

To assess intrahepatocellular DNL, deuterium (10% v/v, CK Isotopes limited, DLM‐2259) was added to experimental media for the entire duration of the treatment (i.e. either 2, 4, or 7 days). Cells were harvested and the TG and phospholipid fractions and quantified and DNL measured as described (Nagarajan et al., 2022).

### 2.6 Confocal microscopy

To assess lipid droplet (LD) size, Huh7 and PHH cells were seeded on 12 mm diameter glass cover slips (VWR, 631‐1577) in 24‐well plates. After the respective treatment period, cells were washed with PBS and then fixed by incubation with 4% paraformaldehyde in PBS for 15 mins. Cover slips were stored in PBS at 4°C until staining. To allow penetration of the stains into cells, cover slips were kept in 0.01% Triton‐X in PBS for 25 mins. Coverslips were then washed twice with PBS and stained with HCS LipidTOX™ Red Neutral Lipid Stain (ThermoFisher, H34476) at 1:200 in PBS and RedDot™2 Far‐Red Nuclear Stain (Biotium, 40061) at 1:125 in PBS for 1 h followed by two more washes with PBS and staining with Oregon Green™ 488 Phalloidin (ThermoFisher, O7466) at 1:40 in PBS for 30 mins. Coverslips were then washed twice with PBS and mounted onto a slide with VECTASHIELD Vibrance® Antifade Mounting Medium (Vector Laboratories, H‐1700) before imaging with 60X magnification on a confocal microscope (Bio‐Rad, Radiance 2100). Images were analyzed using open source CellProfiler4 software.

### 2.7 RNA extraction

RNA was extracted using the RNEasy® Plus mini kit (QIAGEN Sciences, 74,131) according to the manufacturer's instructions. The resulting RNA was treated with DNase (QIAGEN Sciences, 79254) and quality checked with an Agilent Bioanalyzer.

### 2.8 RNA sequencing analysis

Purified RNA samples from 5 donors (see Table S1 for donor characteristics) were sent to Novogene for bulk RNA sequencing. Novogene carried out the initial data processing which involved the following: Removal of low‐quality reads or reads containing poly‐N regions using in‐house perl scripts, indexing and alignment of clean reads to a downloaded reference genome using Hisat2 (version 2.0.5) and finally counting reads mapped to each gene using featureCounts (version 1.5.0‐p3). The following was carried out on all datasets in R (version 4.4.1) on RStudio (version 2024.04.2 + 764). Raw gene count data was filtered to remove genes with low expression, and then normalized using the TMM method (Robinson & Oshlack, 2010) with the EdgeR package (version 4.2.1) (Robinson et al., 2010). FactoMineR and factoextra packages were used to generate principal component analysis (PCA) plots. Differential gene expression analysis using a mixed effects model to control for differences between donors/replicates was performed using the limma package (version 3.60.4) to remove heteroscedasticity from the data (Ritchie et al., 2015). Genes were considered to be differential expressed if the false discovery rate (FDR) <0.05. To determine which pathways were differentially regulated between conditions, gene set enrichment analysis (GSEA) was performed with the package clusterProfiler (version 3.0.4) using the Molecular Signatures Database (MSigDB) Hallmark gene set collection (Liberzon et al., 2015).

### 2.9 Statistical analysis

All experiments were carried out with technical duplicates with at least three independently performed biological repeats. Data are presented as mean ± standard deviation (SD) unless otherwise specified. For comparisons involving two independent groups, the Mann–Whitney *U* test was used. When comparing multiple groups, the Kruskal–Wallis test followed by Dunn's multiple comparisons test was used. Statistical analyses were conducted using GraphPad Prism (version 10). A *p*‐value of <0.05 was considered statistically significant.

## 3 RESULTS

PHH and Huh7 cells were exposed to basal medias and supplements that differed in composition and cells were cultured in treatment media for a different amount of time: 7 days for Huh7 cells and 4 days for PHH cells, which may have influenced the viability and metabolism of cells.

### 3.1 Cell viability

**FIGURE 1:**
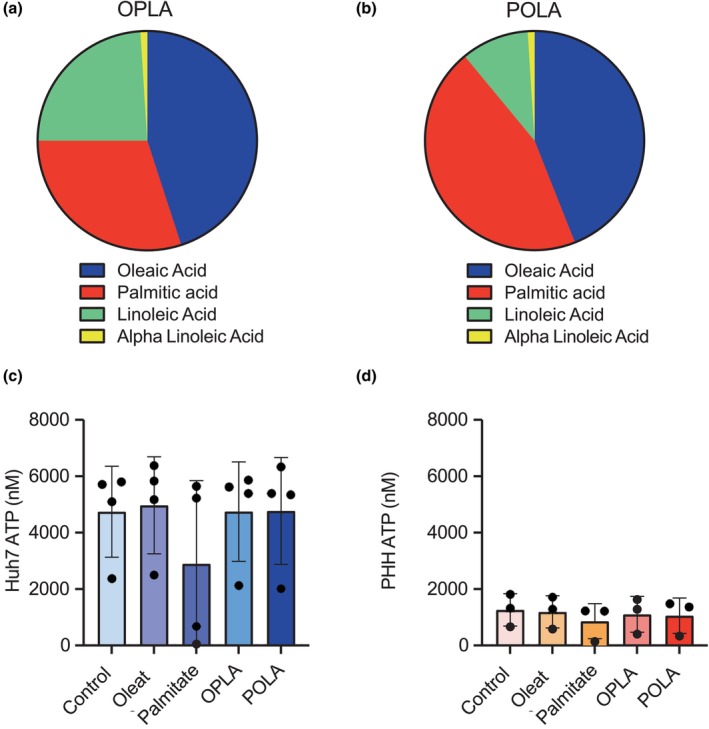
The effects of a single FA and FA mixtures enriched in unsaturated or saturated fatty acids on cell viability. Treatments consisted either of control (no FAs; 5.5 mM glucose), palmitate (400 μM palmitate; 5.5 mM glucose), oleate (400 μM oleate; 5.5 mM glucose), (a) OPLA (800 μM FAs; 5.5 mM glucose) or (b) POLA (800 μM; 5.5 mM glucose) media where FA mixtures were enriched in either unsaturated (O) or saturated (P) FAs. Intracellular ATP was measured as a marker of cell viability in (c) Huh7 (*n* = 4) and (d) Primary Human Hepatocytes (PHH) (*n* = 3) cells. Data are presented as ± SD using Kruskal–Wallis test followed by Dunn's multiple comparisons post‐hoc test for a, b.

When cell viability was assessed, prior to seeding in experimental media, with Trypan Blue, we found 95.6% ± 4.5% of Huh7 cells were viable, whereas only 68.0% ± 9.3% of PHH cells were viable across 5 seeding batches suggesting that prior to seeding PHH cells have lower viability than Huh7 cells. Once seeded, Huh7 and PHH cells were cultured in a single FA (i.e. palmitate and oleate), OPLA (Figure 1a) or POLA (Figure 1b) for 7 and 4 days respectively.

Although we found no significant differences in ATP levels across conditions in both Huh7 and PHH cells, the PHH cells had significantly lower ATP levels across all conditions (Figure 1c,d). There was a higher degree of variability in ATP concentrations of Huh7 cells cultured in palmitate alone compared to other conditions, which was not observed in PHH cells (Figure 1c,d). Our results indicate that Huh7 cells have increased viability compared with PHH cells, and the different cell types may exhibit differing metabolic response in response to culturing with only palmitate.

### 3.2 Fatty acid uptake and media TG

**FIGURE 2:**
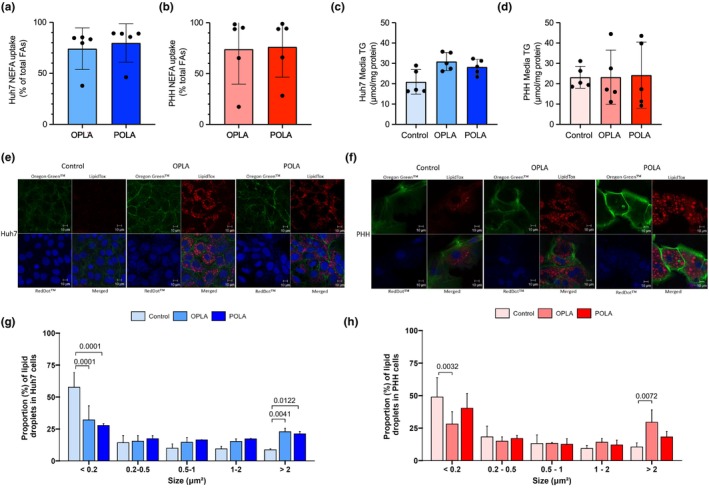
The effects of fatty acid mixtures on fatty acid uptake, secretion and lipid drop size in Huh7 and PHH cells. Non‐esterified fatty acid (NEFA) uptake from the media in (a) Huh7 and (b) PHH cells, media triglyceride (TG) concentrations (c) Huh7 and (d) PHH cells and representative lipid droplet images and size profiling in (e, g) Huh7 and (f, h) PHH cells Data are presented as ± SD (Mann–Whitney *U* test for (a–d); two‐way ANOVA followed by Tukey's HSD multiple comparisons post‐hoc test for (g, h).

Uptake of media NEFA from OPLA and POLA was assessed in Huh7 and PHH cells. In both OPLA‐ and POLA‐treated Huh7 cells, approximately 80% of media NEFA was taken up (Figure 2a). In contrast, in both OPLA‐ and POLA‐treated PHH cells media NEFA uptake was approximately 70% (Figure 2b). Although NEFA uptake was broadly similar between models, greater variability was observed in PHH, consistent with donor heterogeneity (Table S1). Occasional lower uptake values were observed across experiments, highlighting inherent biological and technical variability in PHH cultures. For both Huh7 and PHH cells, there were no significant differences in media TG concentrations between treatments, although there was greater variation within the FA treatments in PHH than Huh7 cells (Figure 2c,d).

### 3.3 Lipid droplet size

The formation of LDs was observed in both Huh7 and PHH cells and appeared greater in OPLA‐ and POLA‐treated cells compared with cells treated with no‐fat control media (Figure 2e,f). These observations were confirmed following image quantification, with both OPLA‐ and POLA‐treated Huh7 cells displaying a larger proportion of LDs >2 μm^2^ and a smaller proportion of LDs <0.2 μm^2^ compared to no‐fat control media treated cells (Figure 2g). In contrast, OPLA‐treated PHH cells had significantly more LDs >2 μm^2^ and fewer LDs <0.2 μm^2^ than PHH cells cultured in no‐fat control media, whereas POLA‐treated PHH cells did not (Figure 2h). There were no significant differences in LD size distribution between OPLA‐ and POLA‐treated Huh7 or PHH cells (Figure 2g,h).

### 3.4 Intrahepatocellular TG accumulation

**FIGURE 3:**
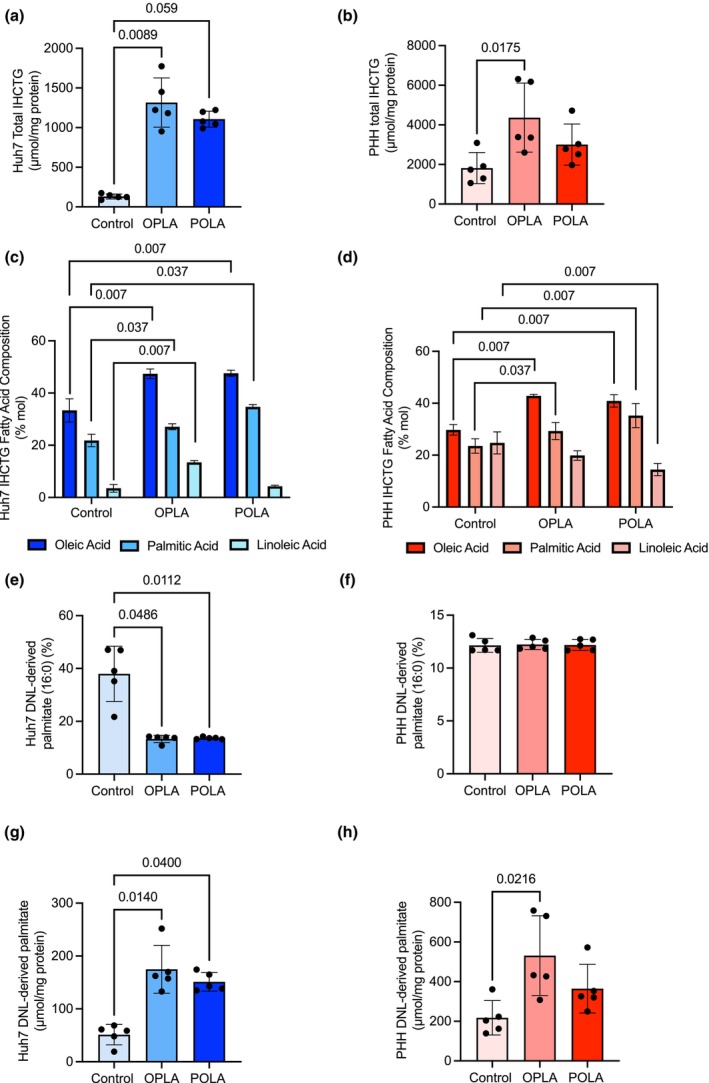
The effects of fatty acid mixtures on intrahepatocellular accumulation and DNL. The intrahepatocellular triglyceride (IHCTG) content of (a) Huh7 and (b) PHH cells, IHCTG fatty acid composition in (c) Huh7 and (d) PHH cells after treatment. The proportion (%) and absolute contribution of DNL‐derived palmitate was assessed in (e, g) Huh7 and (f, h) PHH cells. Data are presented as ± SD (Kruskal–Wallis test followed by Dunn's multiple comparisons post‐hoc test for (a,b, e–h); Mann–Whitney *U* test for (c, d).

In both Huh7 and PHH cells IHCTG content significantly increased with OPLA‐treatment but only tended (*p* = 0.058) to increase with POLA‐treatment compared with no‐fat control treated cells (Figure 3a,b). No‐fat control treated Huh7 cells had minimal IHCTG accumulation compared to PHH cells which had an IHCTG concentration over 20 times more (Figure 3a,b), suggesting PHH cells started with a notably higher IHCTG content than Huh7 cells. This higher baseline lipid burden suggests PHH may retain donor‐specific steatotic characteristics, which could potentially constrain further lipid storage responses in vitro.

We characterized the FA composition of the accumulated IHCTG content in both Huh7 and PHH cells. We found changes in the IHCTG composition of OPLA‐ and POLA‐treated Huh7 cells to reflect the respective FA media composition when compared to the no‐fat control cells (Figure 3c). When compared to no‐fat control cells, the proportions of oleate, palmitate, and linoleate were significantly higher in OPLA‐treated cells, whilst there was no difference between linoleate levels in the no‐fat control and POLA‐treated cells (Figure 3c). Similarly, for PHH cells, those treated with OPLA and POLA had increases in proportions of oleate and palmitate, reflective of the media compositions (Figure 3d). In contrast, linoleate levels in PHH POLA‐treated cells decreased significantly, whereas there was no notable change in OPLA‐treated cells compared to the no‐fat control cells (Figure 3d).

### 3.5 DNL and ketogenesis

We found the percentage contribution of DNL‐derived palmitate to total IHCTG was significantly lower, at around 13% in OPLA‐ and POLA‐treated Huh7 cells compared to no‐fat control cells which were around 38% (Figure 3e). In PHH cells the percentage contribution of DNL‐derived palmitate was similar, at 12%, across all treatments. When we accounted for pool size, we found the amount of DNL‐derived palmitate in IHCTG to be significantly lower in no‐fat control compared to OPLA‐ and POLA‐treated Huh7 cells (Figure 3g), reflecting the greater amount of IHCTG with FA treatment. In PHH cells, although OPLA‐treated cells had a significantly higher contribution of DNL‐derived palmitate, POLA‐treated cells did not when compared to no‐fat control cells (Figure 3h). Although we did not directly compare the contribution of DNL‐derived palmitate to IHCTG between FA treated Huh7 and PHH cells, there was a higher amount in PHH (between 249 and 758 μmol/mg protein) compared to Huh7 (between 132 and 251 μmol/mg protein), which likely reflects the higher IHCTG that PHH cells started with.

**FIGURE 4:**
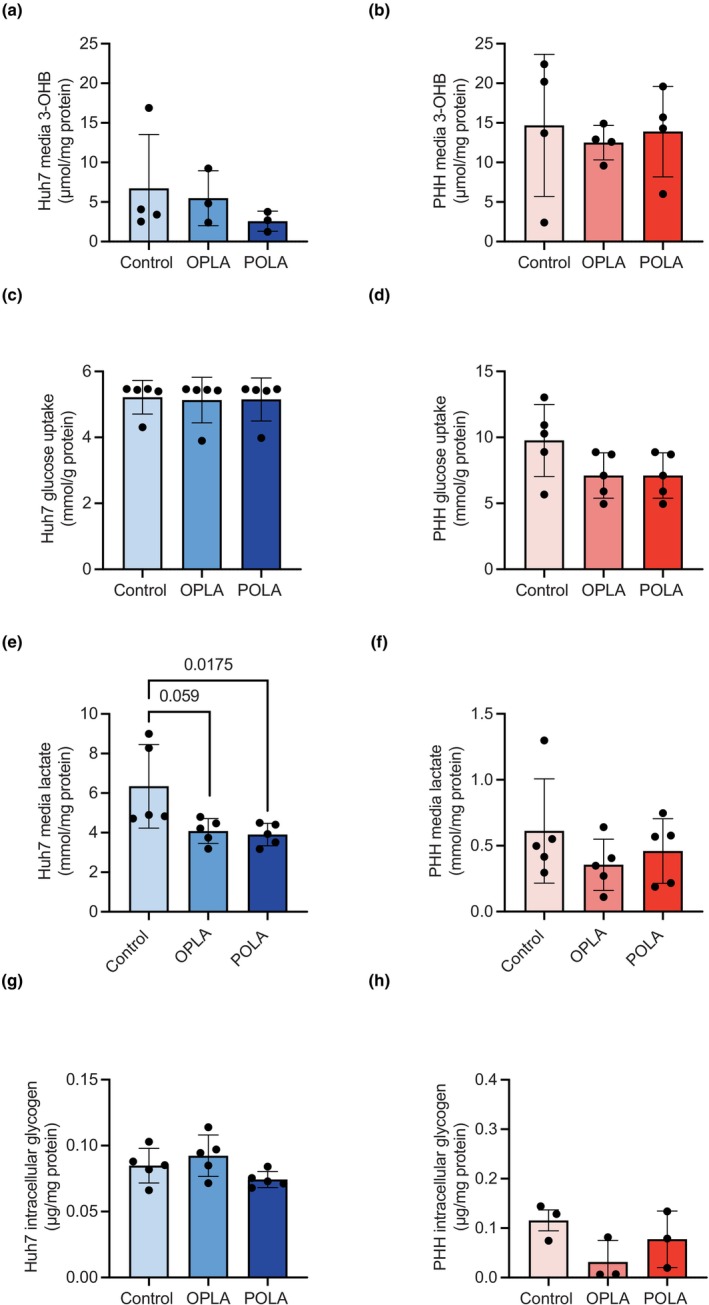
The effects of fatty acid mixtures on 3‐hydroxybutryate concentrations and glucose metabolism in Huh7 and PHH cells. (a) Media 3‐hydroxybutryate (3‐OHB), (c) Glucose uptake, (e) Media Lactate, and (g) intrahepatocellular glycogen concentrations in Huh7 cells, and (b) Media 3‐hydroxybutryate (3‐OHB), (d) Glucose uptake, (f) Media Lactate, and (h) intrahepatocellular glycogen concentrations in PHH cells. Data are presented as ± SD using Kruskal–Wallis test followed by Dunn's multiple comparisons post‐hoc test for (a–h).

Although there were no statistically significant differences in 3‐hydroxybutyrate (3‐OHB) in both Huh7 and PHH cells treated with OPLA, POLA and no‐fat control media (Figure 4a,b), media concentrations appeared to be higher in PHH compared to Huh7 cells.

### 3.6 Glucose uptake, storage, and oxidation

For Huh7 cells cultured in 5mM of glucose, there were no significant differences observed between conditions in the uptake of media glucose between media changes (Figure 4c). PHH cells were cultured in 11mM of glucose, and while there were no significant differences in the absolute glucose uptake between PHH control, OPLA‐ and POLA‐treated cells (Figure 4d), approximately 40% of the available media glucose remained in the media at the end of the culturing period. In contrast, only ~5% of media glucose remained in the Huh7 cell media, consistent with a greater overall utilization of available glucose under these conditions.

Media lactate levels tended (*p* = 0.059) to be lower and were significantly lower in OPLA‐ and POLA‐treated compared to no‐fat control‐treated Huh7 cells, respectively (Figure 4e). In contrast, there were no differences in media lactate between conditions in PHH cells (Figure 4f), which were notably lower than the media lactate levels from Huh7 cells. No significant differences were observed in intracellular glycogen concentrations in both Huh7 and PHH no‐fat control, OPLA‐ and POLA‐treated cells (Figure 4g,h).

### 3.7 RNA Seq data

**FIGURE 5:**
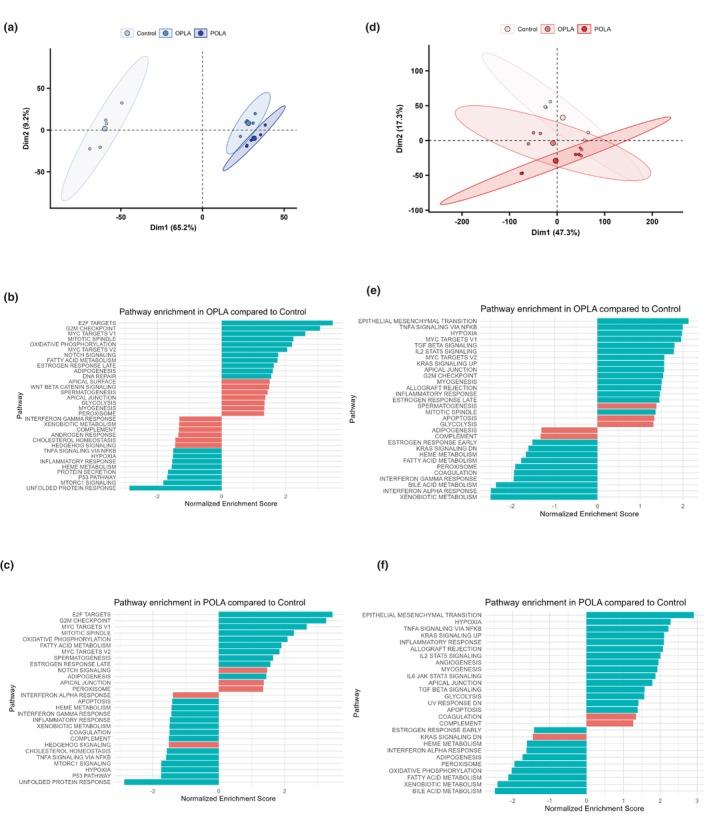
The effects of FA mixtures on gene expression in Huh7 and PHH cells. Principal component analysis plots showing the first two dimensions (dim1 and dim2) for (a) Huh7 and (b) PHHs cells treated with no‐fat control, OPLA or POLA. Gene set enrichment analysis for OPLA‐treated compared to Control‐treated cells in (c) Huh7 and (d) PHH cells and for POLA‐treated compared to Control‐treated in (e) Huh7 and (f) PHH cells. Pathways with the highest and lowest normalized enrichment score are shown with blue pathways *p* < 0.05 and red pathways *p* ≥ 0.05 (GSEA performed with FDR correction for multiple comparisons).

Principal component analysis of Huh7 cell transcriptomic data showed the difference in gene expression between Control and OPLA/POLA conditions accounted for the largest component of the total variance (dimension 1–65.2%, Figure 5a) in the data and the difference in gene expression between OPLA and POLA conditions accounted for the second largest component (dimension 2–9.2%). Gene set enrichment analysis (GSEA) showed that proliferation, oxidative phosphorylation and FA metabolism pathways were the most significantly activated whereas inflammation, unfolded protein response and mammalian target of rapamycin complex 1 (MTORC1) pathways were the most significantly suppressed pathways in both OPLA‐ and POLA‐treated Huh7 cells compared to no‐fat control‐treated Huh7 cells (Figure 5b,c). When comparing Huh7 cells treated with OPLA and POLA, pathways associated with proliferation were upregulated with POLA treatment and complement, coagulation and cholesterol homeostasis pathways were upregulated with OPLA treatment (Figure S1a).

In contrast, the largest component of total variance in the PHH transcriptomic dataset was driven by a difference in gene expression between donors (dimension 1–47.3%, Figure 5d). However, the second largest component of total variance appeared to be the difference in gene expression between no‐fat control and OPLA/POLA conditions (dimension 2–17.3%). GSEA highlighted the activation of inflammatory pathways and epithelial mesenchymal transition and the suppression of FA and bile acid metabolism in both OPLA and POLA conditions when compared to no‐fat control (Figure 5e,f). Comparison between PHH cells treated with OPLA and POLA showed inflammatory and coagulation pathways upregulated with POLA treatment and proliferation, oxidative phosphorylation, and FA metabolism pathways upregulated with OPLA treatment (Figure S1b). These findings indicate that, in contrast to Huh7 cells, PHH transcriptional responses to FA exposure are strongly influenced by donor‐specific baseline differences.

## 4 DISCUSSION

There is currently a lack of high‐fidelity models, particularly in vitro cellular models, that recapitulate FA and glucose metabolism, along with IHCTG accumulation observed in early stages of human MASLD. Although PHH are considered to be the gold standard for in vitro hepatocellular models, hepatoma cell‐lines (i.e. Huh7 and HepG2) appear to be used more routinely as an experimental model. Data comparing PHH and cell‐lines as a model to interrogate MASLD initiation and progression is sparse, therefore we compared the metabolic responses of PHHs with our Huh7‐cell model of IHCTG accumulation (Nagarajan et al., 2022) using chronic (4–7 days) of exposure to physiologically relevant substrates. Despite PHH starting with a higher amount of IHCTG than Huh7 cells, we observed similarities in key metabolic responses including NEFA uptake and IHCTG FA composition, media TG concentrations (reflecting TG secretion), LD morphology, DNL responses, and glycogen levels. Notable differences in responses included PHH having lower and more variable viability than Huh7 cells, higher media 3OHB and lower media lactate concentrations, along with divergence in transcriptomic datasets which, for PHH cells was mostly driven by the gene expression between donors. Together, these data show that Huh7 cells cultured in physiologically relevant media reproduce key lipid‐handling phenotypes observed in PHH cells, supporting their use as a scalable workhorse model for mechanistic studies of early IHCTG accumulation. However, PHH remain essential for capturing donor‐depended variability and inflammatory stress responses relevant to MASLD progression.

IHCTG accumulation is suggested to be due to an imbalance between input, synthesis and disposal pathways (Smith et al., 2025). It is well established that exposing hepatocytes to increasing concentrations of dietary FAs in vivo (Luukkonen et al., 2018; Parry et al., 2020; Rosqvist et al., 2014, 2019) or media FAs in vitro (Breher‐Esch et al., 2018; Gunn et al., 2020; Nagarajan et al., 2022) can lead to an increase in IHCTG accumulation. In line with previous in vitro studies that have tended to culture hepatocytes short‐term (<48 h) in a single FA or mix of FA (Breher‐Esch et al., 2018; Liu & Green, 2019; Maruyama et al., 2014; Moliterni et al., 2024; Wei et al., 2006), we found both PHH and Huh7 cells accumulated IHCTG with FA treatment compared to the no‐fat control. The increase in IHCTG was more evident in Huh7 than PHH cells, despite no notable differences in FA uptake or the FA composition of IHCTG between cell types as both PHH and Huh7 reflected the respective media FA composition after treatment. The lower accumulation of IHCTG by PHH cells may in part be explained by the cells having approximately 4 times more IHCTG than Huh7 cells, suggesting a more limited capacity for IHCTG storage. We assessed the change in IHCTG FA composition compared to the no‐fat control cells and found for both PHH and Huh7, it reflected the composition of the FA treatment. There was a notable decrease in linoleate in POLA‐treated PHH cells, which is likely due to the dietary fat the PHH donors were exposed to in the days prior to hepatocyte isolations, being turned over and replaced with the FA in the culture media, where the linoleate content of POLA was only 10%.

Previous work has shown that Huh7 cells cultured with palmitic acid accumulate less IHCTG than those cultured with oleic acid (de Sousa et al., 2021; Eynaudi et al., 2021; Gomez‐Lechon et al., 2007; Lee et al., 2010; Listenberger et al., 2003; Ricchi et al., 2009), which may be due to oleic acid promoting storage of FAs in LDs (Moravcova et al., 2015) and palmitic acid activating pro‐apoptotic pathways (Liu & Green, 2019; Maruyama et al., 2014; Moliterni et al., 2024; Wei et al., 2006). Work by Teixeira and colleagues (Teixeira et al., 2023) demonstrated that when HepG2 cells are cultured in 1 mM of palmitic acid, linoleic acid or a 1:1 ratio of the two, IHCTG content was highest when cells were cultured with linoleic acid alone, or in combination with palmitic acid. As we wanted to make our media physiologically relevant, we chose to utilize a mixture of FAs that reflect the major dietary FAs and those found in the human body (Hodson et al., 2008) and manipulated the proportions of saturated and polyunsaturated FAs. We compared our FA treatments to the more historically used single FA treatments of palmitate and oleate and found that palmitate treatment alone resulted in higher variability in ATP levels in Huh7 cells, a pattern not observed in PHHs. Our findings suggest that even though PHH had lower viability than Huh7, the use of mixed FA chronically (4–7 days) reduced any confounding effects associated with acute lipotoxicity (Liu & Green, 2019; Wei et al., 2006). We cultured both Huh7 and PHH cells in 2% HS, which may have exposed cells to greater than anticipated insulin concentrations. When measured, we found the insulin concentration in the media that cells were cultured in was 0.00072 nmol/L and we added 0.10 nmol of insulin to our media. We acknowledge that although HS contains insulin, we believe, although cannot exclude, that the additional amount of 0.00072 nmol/L will have had minimal impact on cellular metabolism. We did not measure IGF‐1 concentrations in HS, but it would be of interest to do so. Thus, media composition can have major effects on the validity, reproducibility and physiological relevance of the scientific findings; therefore, choosing an appropriate cell culture medium matters, particularly in metabolic studies (Lagziel et al., 2020).

Although we found PHH cells to have a higher amount of IHCTG than Huh7 cells, we did not observe notable differences in proportions of LD sizes across the two cell types. In Huh7 cells, IHCTG content increased with FA treatments, as did the LD size, whilst in PHH cells only culturing in OPLA, compared to POLA induced a significant increase in IHCTG content and the proportion of larger LDs. Kwon et al. (Kwon et al., 2024) cultured PHH cells in a 3D collagen sandwich for up to 7 days with a FA mix of palmitic and oleic acids (ratio 1:5) in varying concentrations and noted an increase in LD size with higher concentrations of FAs. Paramitha and colleagues (Paramitha et al., 2021) used Raman spectroscopy to compare the effects of specific FA on LD size in HepG2 cells, found the average LD size increased between Day 1 and 5 by 11%, 41%, and 78% in cells cultured in media, which was changed every 24 h, containing 100 μmol of palmitic, oleic or linoleic acid, while average LD number per cell increased by 58%, 599%, and 233%, respectively. Although we did not assess the number of LDs, it is plausible there was a greater increase in the number of LDs with IHCTG accumulation in Huh7 compared to PHH cells, where there was likely a more notable change in LD number in cells cultured in unsaturated compared to saturated FAs. As LDs are complex, metabolically active organelles in hepatocytes, a notable increase in number and size may cause hepatocyte ballooning and at a whole organ level, lead to anatomical changes in the liver (Scorletti & Carr, 2022) which may lead to metabolic dysregulation at the whole‐body level. Thus, understanding the mechanisms governing LD number and size, along with the impact FA composition may have, will add insight for the further development of therapeutic strategies.

An upregulation in hepatic DNL is a hallmark of insulin resistance and MASLD and has been suggested to promote IHCTG accumulation (Solinas et al., 2015). We found the percentage contribution of DNL‐derived palmitate to IHCTG palmitate to be similar in both cell types with FA‐treatment and comparable with what we have previously reported for fasting DNL, in vivo in metabolically‐healthy humans (Lambert et al., 2025; Luukkonen et al., 2023; Pramfalk, Pavlides, et al., 2016; Smith et al., 2020). For the no‐fat control treated cells, there was a higher percentage contribution in Huh7 compared to PHH cells, with the latter being similar to the FA‐treated PHH and Huh7 cells. After accounting for differences in IHCTG palmitate pool size between the cells, the contribution of DNL‐derived palmitate was greater in PHH, reflective of the larger amount of IHCTG pool compared to Huh7 cells. It is also plausible that given the phenotype of the donors and respective intakes of alcohol, DNL was an upregulated process (Siler et al., 1999) and the PHH cells had a higher proportion of DNL‐derived palmitate in the IHCTG at seeding.

When glucose is consumed in vivo in humans, there is a corresponding insulin response which helps co‐ordinate the utilization of glucose to minimize fluctuations in glycaemia by providing a signal for hepatic glycogen deposition, and upregulating the hepatic DNL pathway (Cross et al., 2023). Previous work by Nagarajan and colleagues identified clear divergences in insulin‐stimulated metabolism between primary mouse hepatocytes and immortalized hepatoma HepG2 cells (Nagarajan et al., 2019). We found glycogen deposition to be similar between both cell types and across all treatments, which may in part be explained by the fact that we did not add insulin into the media and therefore did not upregulate glycogen deposition; it would be of interest to see if this pathway could be upregulated in future experiments.

For Huh7 cells, the lack of excess substrate, due to being cultured in lower amounts of glucose than PHH, may have contributed to the lower levels of intracellular glycogen. Alternatively, any excess glucose may have been partitioned toward glycolysis as media lactate levels were notably higher in Huh7 compared to PHH cells under all treatments, which is likely due to Huh7 being a hepatoma‐derived cell line and has been reported to rely on glycolysis for rapid growth (Kiss et al., 2025).

The divergence in the media concentrations of lactate and 3OHB as markers of FA and glucose oxidation by PHH and Huh7 cells was one of the more notable differences between the cell types. This distinction may be particularly relevant for modeling MASLD where altered hepatic substrate oxidation and ketogenesis are key early metabolic adaptations. In line with our current observation of a significant decrease in media lactate with the addition of FAs, we have previously found in Huh7 cells that media lactate is significantly lower when cells were cultured in 800 μM FA and 11 mM glucose + 5.5mM fructose when compared to cells cultured in 200 μM FA and 11 mM glucose + 5.5 mM fructose (Gunn et al., 2020). Thus, it would be of interest to culture Huh7 longer in lower glucose and higher FA to see if they metabolically shifted away from glycolysis toward greater FA oxidation. The ketone body, 3OHB, is often used as a surrogate marker of hepatocellular FA oxidation and we found that media concentrations were approximately 3‐fold lower in Huh7 compared to PHH cells, with no differences being noted across treatments. We have previously found media 3OHB to increase when Huh7 cells were cultured in 800μM FA and 11mM glucose + 5.5mM fructose compared to cells cultured in 200 μM FA and 11mM glucose + 5.5 mM fructose (Gunn et al., 2020). The higher media 3OHB in PHH may in part explain the lower magnitude of increase in PHH IHCTG compared to Huh7 with FA treatment, with some of the excess FA being disposed via oxidation. Although we did not measure complete mitochondrial oxidation, we have previously found Huh7 cells cultured in 800 μM FA and 5.5mM glucose for 7 days had higher FA oxidation compared to cells cultured in 200 μM and 5.5mM glucose (Nagarajan et al., 2022). The availability of FA to enter oxidation pathways may be influenced by how rapidly LDs turnover and FA are made available; it remains to be elucidated if differences in LD TG turnover exist between FA treatments and cell types.

Transcriptomic analysis revealed both overlap and divergence in responses of PHH and Huh7 cells treated with FA. In Huh7 cells, the dominant source of transcriptional variance was FA treatment, whereas in PHH cells transcriptomic variability was driven primarily by donor‐specific differences, suggesting that the genotype and baseline phenotype of these cells have a far greater impact on transcription than treatment with FAs. Transcriptomic analysis suggested that Huh7 cells become physiologically adapted to these FA mixtures after chronic exposure with a shift toward oxidative phosphorylation and FA metabolism with reduced stress and inflammatory responses. However, this muted inflammatory response in Huh7 cells likely reflects their transformed origin and reduced competence for stress and immune signaling, meaning PHH remain essential for modeling inflammatory aspects of MASLD progression. In contrast, PHH cells displayed activation of stress‐related pathways with FA treatment, together with suppression of lipid metabolic pathways. This divergence highlights a fundamental biological difference between Huh7 and PHH cells in modeling aspects of FA‐induced hepatocellular stress.

The absence of inflammatory pathway activation in Huh7 cells may reflect their transformed origin and altered stress‐response signaling, whereas the inflammatory transcriptional response observed in PHH cells is likely influenced by higher baseline intracellular lipid content and reduced cellular viability following cryopreservation. These factors may also constrain the dynamic range of transcriptional responses in PHH and contribute to the observed differences between OPLA‐ and POLA‐treated conditions across cell types. Responses may also reflect, in part, culture media, duration of FA exposure, along with how long IHCTG had been accumulating, as PHH cells have been exposed to more than FAs and are likely further along the liver disease pathology pathway.

Cells used in in vitro experiments should have high viability, indicating they are live, healthy, and functional, so the effect of a treatment or specific conditions can be robustly assessed. Typically, viability assays assess membrane integrity, metabolic activity, or enzymatic function. We assessed cell viability in two ways, using trypan blue prior to seeding, where cells with compromised membranes take up the dye, and by measuring ATP levels after cells had treatment media for 4–7 days as a proxy for cellular energy state. For both assays, PHH demonstrated lower viability compared with Huh7 cells. These observations are consistent with prior reports that highlight the reduced proliferative capacity and metabolic stability of PHH post‐cryopreservation (Sun et al., 2023), along with the variability in viability noted in the certificate of analysis that is provided with the PHH when purchased.

### 4.1 Limitations of this study

Our study is not without limitations. We used trypan blue staining at seeding to determine cell viability and found it to be lower in PHH cells. As PHH were cultured in collagen‐based adherence methods, non‐viable cells could not be removed and may therefore have contributed to downstream measurements potentially leading to an underestimation in cell viability. Culture duration differed between the two cell types, with PHHs, due to lower viability, being maintained for only 4 days (media changes every 2 days); thus, limiting our ability to explore chronic FA exposure, and Huh7 cells were cultured for 7 days (media changes every 2 days). In addition, basal media composition differed between models, including glucose concentration, which may have influenced substrate utilization and metabolic fluxes. Donor metabolic history prior to hepatocyte isolation is not fully known, which may contribute to baseline lipid variability and transcriptional differences observed in PHH. Therefore, in future studies, where possible, we would culture both cell types for the same duration under, where possible, similar substrate conditions. We assessed intrahepatocellular FA oxidation by measuring the media concentration of 3‐OHB. It would be of interest to assess complete mitochondrial FA oxidation through the use of stable‐isotope tracers, as we have previously done (Nagarajan et al., 2022) along with assessing mitochondrial respiration in both cell types to determine if the divergent responses observed for 3‐OHB are evident. We assessed the difference between the cell types in a 2D model. It would be of interest to compare Huh7 and PHH cells in 3D models, as Bonanini and colleagues (Bonanini et al., 2024) observed higher net glucose production in 3D liver organoids (derived from male donor) compared to HepG2 cells in 2D. Therefore, it would be of great interest to use our media with a physiologically relevant three‐dimensional culture system as such an approach may better capture hepatic zonation, oxygen gradients, and intercellular interactions that influence hepatocyte metabolism in vivo.

## 5 CONCLUSION

Overall, our data indicate that while immortalized hepatic cell lines are often considered to be a poor surrogate for PHH metabolism, Huh7 chronically cultured in physiologically relevant media are metabolically adaptable and show comparable responses that overlap with PHH in selected lipid and metabolic readouts. In particular, similarities were observed in FA uptake, IHCTG composition, and LD characteristics where cells were exposed to physiological levels and composition of FA. These shared features coexist with clear biological differences between the two models. PHH displayed higher baseline IHCTG content and greater donor‐dependent variability, where Huh7 showed more uniform metabolic and transcriptomic adaptation to chronic FA exposure. Taken together, our findings suggest that Huh7 are metabolically adaptable, and with further optimization, they could better recapitulate PHH metabolism, which is dependent on the phenotype and genotype of the donor, thus making Huh7 a practical and scalable workhorse model to investigate relevant pathways that may underpin the development of MASLD.

## AUTHOR CONTRIBUTIONS

**Eloise Cross:** Conceptualization; data curation; formal analysis; investigation; methodology. **Felix Westcott:** Conceptualization; data curation; formal analysis; investigation; methodology. **Kieran Smith:** Formal analysis. **Shilpa R. Nagarajan:** Data curation; formal analysis. **Fabio Sanna:** Methodology; supervision. **Kaitlyn, Margaret‐Joyce Hubay Dennis:** Formal analysis. **Leanne Hodson:** Conceptualization; data curation; formal analysis; funding acquisition; investigation; methodology; project administration; resources; software; supervision; validation; visualization.

## FUNDING INFORMATION

This work was supported by the British Heart Foundation (Fellowship FS/15/56/31645 and FS/SBSRF/21/31013 to L.H.), the Oxford BHF CRE (RE/18/3/34214), the Kennedy Trust Oxford MB PhD Programme (F.W.), and the Biotechnology and Biological Sciences Research Council Institute Strategic Programme Food Innovation and Health (BB/R012512/1 and its constituent project BBS/E/F/000PR10347 to L.H.) and a Novo Nordisk Postdoctoral Fellowship run in partnership with the University of Oxford (S.R.N).

## CONFLICT OF INTEREST STATEMENT

The authors declare no conflict of interest.

## ETHICS STATEMENT

The manuscript contains no data or description of human patients or animals; all the described work has been undertaken using Huh7 and primary human hepatocyte cells.

## Supporting information


**Figure S1.** Gene set enrichment analysis for OPLA compared to POLA treated (a) Huh7 cells and (b) Primary Human Hepatocytes. Pathways with the highest and lowest normalized enrichment score are shown with blue pathways *p* < 0.05 and red pathways *p* ≥ 0.05 (GSEA performed with FDR correction for multiple comparisons).

**Table S1.** Primary human hepatocyte donor characteristics.

## Data Availability

RNA sequencing Data are available and deposited on GEO (GSE334407).
