# Supplementary figures and images for: Physiologically relevant media are associated with overlapping metabolic responses in primary human hepatocytes and Huh7 cells
Source: Physiol Rep. 2026 Jul 3;14(13):e70989. doi: 10.14814/phy2.70989 (PMC13330593; doi:10.14814/phy2.70989)

Supplementary Figure 1.

A.

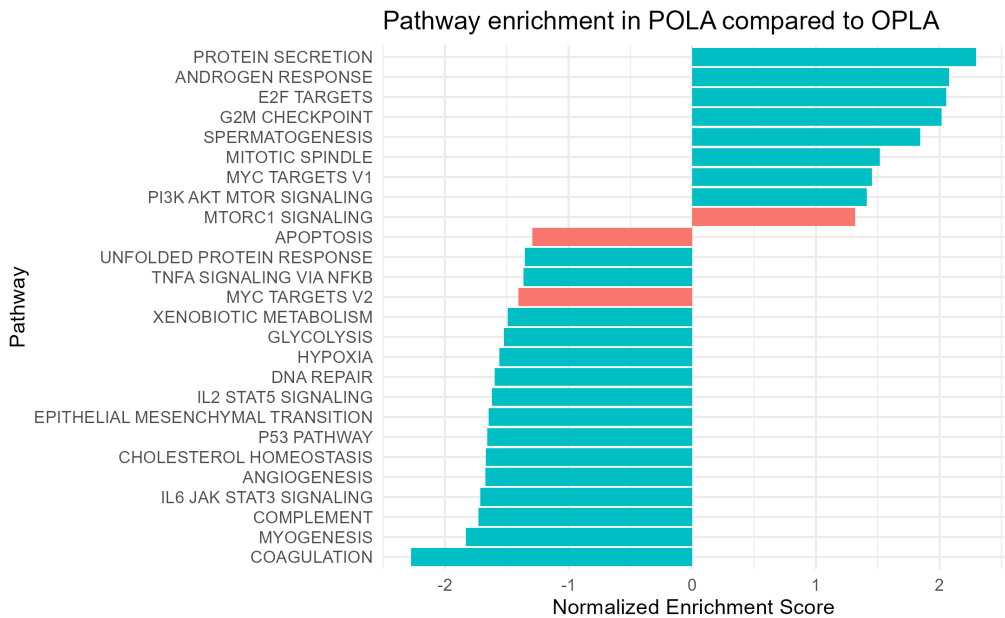

B.

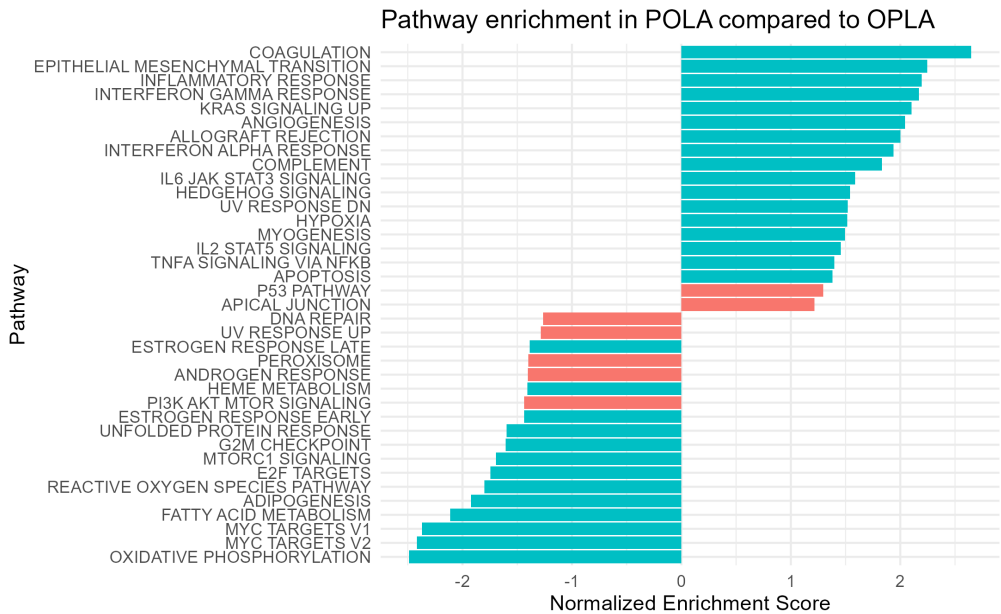

Supplement: Supplementary file 1 — Figure S1. Gene set enrichment analysis for OPLA compared to POLA treated (a) Huh7 cells and (b) Primary Human Hepatocytes. Pathways with the highest and lowest normalized enrichment score are shown with blue pathways p < 0.05 and red pathways p ≥ 0.05 (GSEA performed with FDR correction for multiple comparisons). [file PHY2-14-e70989-s002.pdf]
